# Harm reduction program and hepatitis C prevalence in people who inject drugs (PWID) in Iran: an updated systematic review and cumulative meta-analysis

**DOI:** 10.1186/s12954-020-00441-9

**Published:** 2021-01-22

**Authors:** Abdolhalim Rajabi, Heidar Sharafi, Seyed Moayed Alavian

**Affiliations:** 1 Baqiyatallah Research Center for Gastroenterology and Liver Diseases (BRCGL), Baqiyatallah University of Medical Sciences, Tehran, Iran; 2 Environmental Health Research Center, Faculty of Health, Golestan University of Medical Sciences, Gorgan, Iran; 3 Middle East Liver Diseases (MELD) Center, Tehran, Iran

**Keywords:** HCV, Intravenous drug use, Harm reduction, Iran

## Abstract

**Background:**

Prevalence of hepatitis C virus (HCV) infection among people who inject drugs (PWID) in Iran is high. Since 2005, the Iranian government has implemented a harm reduction program to control HCV. We aimed to describe the prevalence of HCV antibody (Ab) in Iranian PWID before and after the implementation of harm reduction with cumulative meta-analysis.

**Methods:**

Following PRISMA guidelines, we conducted a systematic review and meta-analysis of studies published on the seroprevalence of HCV among PWID. We systematically reviewed the literature to identify eligible studies up to December 2018 in international and national databases. Pooled prevalence and 95% confidence intervals were calculated using Der Simonian and Laird method, taking into account conceptual heterogeneity. Subgroup analyses were performed by harm reduction implementation and studies’ characteristics to assess the sources of heterogeneity. We used Cochran–Armitage test for the linear trend of the prevalence of HCV Ab among PWID.

**Results:**

We reviewed 5966 papers and reports and extracted data from 62 eligible records. The pooled HCV Ab prevalence among PWID in Iran was 46.5% (95% confidence interval [95% CI] 41.1–52.0%). Overall, the Cochran–Armitage test for trend indicated a significant decreasing trend of HCV Ab prevalence (*P* = 0.04). The cumulative meta-analysis showed a slight decline in the prevalence of HCV Ab between the years 2005 and 2018.

**Conclusions:**

The HCV Ab prevalence among PWID in Iran is high, with a considerable geographical variation. The prevalence of HCV Ab among PWID in Iran slightly decreased after 2005 which could be, at least to some extent, related to the implementation of extensive harm reduction programs in the country.

## Introduction

Hepatitis C virus (HCV) infection is the main global health concern. The global prevalence of viraemic HCV infection is estimated to be 1% in 2015, corresponding to 71.1 million (62.5–79.4) viraemic infections [1]. Evidence shows the incidence of three to four million people each year, and millions of people with chronic infection are at risk of developing cirrhosis and liver cancer [2]. HCV is transmitted widely through injection of the drug; however, unsafe medical injections were the predominant route of transmission in developing countries [3]. No effective HCV vaccine is yet available. Therefore, public health interventions are the only means of preventing HCV, especially in high-risk populations. People who inject drugs (PWID) are particularly at risk for contracting HCV and other blood-borne diseases, including those caused by human immunodeficiency virus (HIV) and hepatitis B virus (HBV) [4–6]. Opioid substances are associated with the highest drug-related burden [7, 8]. Injecting drug use has also expanded in the world in the last decades, and it is estimated that 15.6 million people inject drugs worldwide [9]. According to a Harm Reduction International report in 2013 and other sources, it is estimated that there are 170,000 to 230,000 PWID in Iran [10–13]. Opium has traditionally been used in Iran, but rapid changes in drug use patterns have occurred in recent decades, leading to an increased number of PWID [14, 15]. Iran has one of the highest numbers of people who use drugs (PWUD); many of them have a history of drug injection [14–16]. In Iran, a history of drug use, especially drug injection, is the main risk for transmission of HCV [17, 18]. World Health Organization has set a goal for the elimination of HCV by 2030 which needs continuous harm reduction for containment of HCV epidemics accompanied by “test and treat” initiatives in the target populations such as PWID (HCV micro-elimination) in Iran [19, 20]. More specifically, the prevalence of HCV antibody (Ab) among PWID in Iran has been reported 40–75%, depending on the study location [21]. A systematic review of PWID between 2000 and 2016 showed that the prevalence of HCV Ab in PWID in Iran was 42% (95% CI 33–52%) [22].

In Iran, during the end of 1990s and early 2000s, drug injection was common and general knowledge of PWUD on blood-borne infections was low [23, 24]. In 2002, Iran implemented a harm reduction program including opioid substitution treatment (OST), needle and syringe programs (NSP), outreach, and prison-based programs, primarily in response to the HIV epidemics among PWID [25]. These programs included education, OST by methadone and buprenorphine, and ensuring access to sterile syringes and needles, as well as condoms [25]. The need for a harm reduction program originated in a survey of 900 street drug users which found that the HIV prevalence was 25% among PWID [26]. These programs have been scaled up at the national level of Iran and assigned as an official policy since 2005 [25, 27]. The Iranian Ministry of Health reported that in 2007, 654 centers provided methadone maintenance therapy (MMT) and buprenorphine maintenance therapy (BMT) to 20,000 and 2,000 opioid dependents, respectively. In prisons, 11,000 prisoners were receiving MMT [28]. According to the report of the Ministry of Health, in the year 2011, thousands of methadone clinics, mainly in the private sector, were providing OST and about 500,000 PWUD were receiving OST. Moreover, tens of thousands of prisoners were receiving MMT [29]. About the needle and syringe program, in 2007, a total of 120 drop-in-centers (DICs) and 150 outreach teams distributed 1,400,000 needles and syringes [28]. In mid-2011, hundreds of sites were providing needles and syringes, as well as condoms, and in 1 year, millions of syringes were distributed [30-32]. Although there is sufficient evidence on the effectiveness of NSP and OST in the reduction in self-reported injecting risky behavior [32–34], evidence regarding the effectiveness of these programs in the containment of HCV epidemics is inadequate in Iran [32, 33].

Previously, a systematic review had been conducted on the HCV prevalence among PWID in Iran, and the included studies were conducted from 2000 through 2016 [22]. The current systematic review was conducted to: (1) provide an updated estimate of the HCV Ab prevalence among PWID in Iran; (2) estimate the HCV Ab prevalence in study settings and geographical locations; (3) analyze the trend of HCV Ab prevalence over time; and 4) compare the HCV Ab prevalence before and after the implementation of harm reduction program in Iran.

## Methods

An extensive and comprehensive search was done in the international (PubMed, ISI Web of Science, Scopus, Embase), regional (IMEMR), and national (Barakat Knowledge Network System) databases in December 2018. To access studies not yet published by that date, expert authors in this field were contacted. Moreover, the reference lists of the studies included in the final analysis as well as previous systematic reviews conducted in Iran were reviewed [22, 35]. In this review, PWID was defined as people who have injected illicit drugs at least once during the past 12 months. The methods used were following the PRISMA [36] and GATHER guideline [37] (Checklist presented in Appendix 1). The protocol was registered in PROSPERO (CRD42018104303).

### Search strategy

A broad search was conducted using the MeSH terms and text words (and their combinations and truncated synonyms) of geographical location (i.e., country and province names) and HCV in electronic data sources including PubMed, EMBASE, Education Resource Information Center, MEDLINE, MEDLINE in process, PsycINFO, Scopus, and Web of Science, Iranmedex, Google Scholar, Iranian Data Bank of Hepatitis Research, Scientific Information Database (SID), Magiran, and the Iran Blood Transfusion Journal. In our search strategy, we did not apply any limitations in the time of publication and language. Iranian databases were searched using the related keywords and the Persian equivalents of HCV, considering all possible combinations. We reviewed the titles and abstracts to select potentially relevant papers. If there was doubt about the suitability of the paper based on the abstract, the full text was reviewed. We manually searched the references and relevant articles for inclusion. We also looked at the electronic abstract list of congresses conducted in Iran and also at the electronic database of students’ thesis through universities’ electronic libraries and Web sites. Furthermore, we searched and identified studies that were not captured by our database by reviewing the previously published meta-analyses and the reference lists in retrieved articles [22, 35, 38]. The search strategy is presented in Appendix 2.

### Study selection

**Fig. 1:**
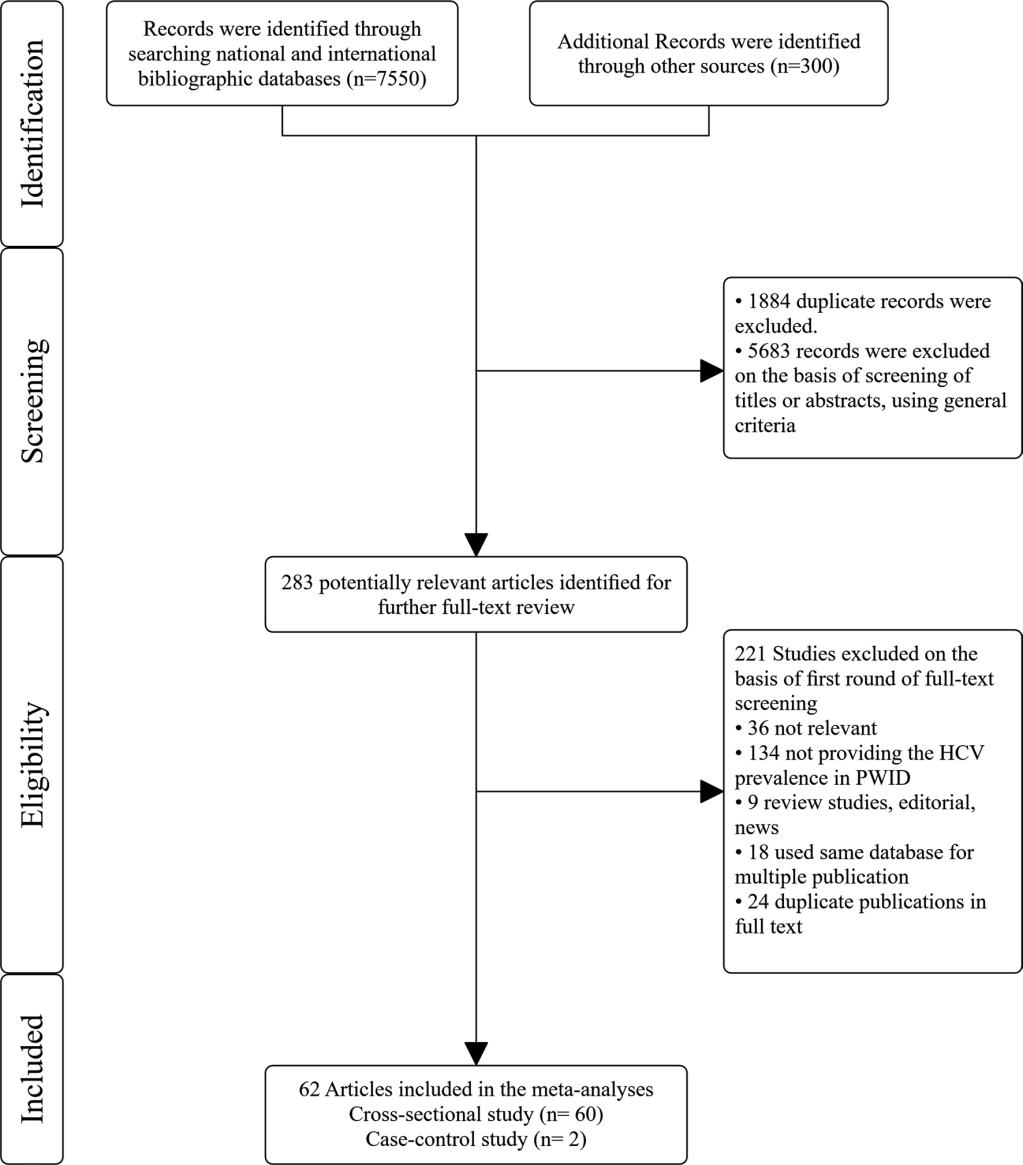
Flowchart of the search and selection process

All records identified through our search were imported into an EndNote library where duplicate publications were identified and excluded (Fig. 1). Similar to our previous systematic reviews [39], the remaining unique reports underwent two stages of screening, performed by AR and HSH. The titles and abstracts were first screened, and those deemed relevant or potentially relevant underwent further screening, in which the full texts were retrieved and assessed for eligibility, based on our inclusion and exclusion criteria. Eligible reports were included in this study, while the remaining ineligible reports were excluded for reasons indicated in Fig. 1. The references of all full-text articles and literature reviews were also screened for further potentially relevant reports.

### Eligibility criteria

The objective of screening was to identify the scientific documents with epidemiological evidence on the prevalence and/or incidence of HCV Ab among PWID. The following five eligibility criteria were used in the selection of relevant studies: document type, study design, disease area, geographical setting, and population.

Concerning the document type, all scientific documents reporting original data (i.e., gathered directly by conducting surveys and laboratory tests on specimens), in the form of a peer-reviewed manuscript, progress report, abstract, technical report, or substantive scientific commentary, were included. Documents not reporting epidemiological data (e.g., legal cases, legislation) and those not reporting original data (e.g., data simulation), as well as documents lacking scientific and methodological details needed for the assessment of the validity of findings (e.g., media reports), were excluded. Documents that were self-described as “systematic reviews” or “meta-analyses” (scientific documents such as peer-reviewed manuscripts or abstracts summarizing results from a group of epidemiological studies) were retained for hand searching of the references. All study designs reporting data on the prevalence and incidence of disease, including cross-sectional studies, cohort studies, population case–control studies, and even experimental studies (e.g., randomized controlled trials) in which a biological survey was conducted to test for HCV before the introduction of the intervention (baseline) were included. Case reports, case series, and qualitative studies were excluded. For the disease area, studies with biological evidence on infection with HCV were included. Self-reported data on the diseases were excluded. The geographical scope was limited to studies conducted within Iran; the studies conducted among Iranian populations residing outside of Iran were not included. For the study population, studies conducted in human subjects who self-identified as current or former drug users were included. Studies on populations who were in short-term mandatory drug treatment and rehabilitation detention centers were included. Studies in non-human subjects, blood donors, dialysis patients, pregnant women, families or sexual partners of HCV/HBV patients, and populations with other chronic diseases (e.g., studies reporting HCV among people with liver cancer) were excluded. No limits were set on study implementation or publication date.

### Data extraction, quality assessment, and risk of bias

Two authors (AR and HSH) reviewed the retrieved studies independently, and the following information was extracted: name of the first author, year of publication, date of study, location of study, total sample size, the prevalence of HCV Ab, recruitment setting, and recruitment method. The third author (SMA) was determined as the arbiter to resolve any disagreements.

Two independent investigators (AR and HSH) assessed the quality of the studies using the Critical Appraisal Skills Programme (CASP) tools for observational studies [40] and a critical appraisal tool for prevalence studies [41]. We used the following criteria for critical appraisal: (1) sampling method; (2) recruitment setting; (3) presenting the type of test; (4) presenting the definition of PWID; and (5) refusal rate. The documents were evaluated in terms of the above-mentioned criteria. Discrepancies were resolved by discussion or through consultation with the third investigator (SMA).

### Data analysis

The extracted data were entered into the Excel software. Then, Stata 16.0 was used for analysis. Pooled prevalence and 95% confidence intervals were calculated using Der Simonian and Laird method, taking into account conceptual heterogeneity, and I^2^, Tau^2^, and X^2^ were applied to assess heterogeneity among studies. The “metaprop” command was used to calculate the pooled prevalence of HCV Ab and the prevalence in different subgroups, by available geographical locations and study settings. The pooled prevalence of HCV Ab before 2005 (before scale-up of the harm reduction program at the national level) and during and after 2005 (after scale-up of the harm reduction program at the national level) was presented in a forest plot, and the heterogeneity of studies conducted in each period was estimated. The Q test was applied to assess heterogeneity between periods. Furthermore, the presence and the effect of publication bias were investigated using a combination of the visual inspection of funnel plots and Begg’s and Egger’s tests. Moreover, a trim-and-fill analysis was performed to assess the stability of overall prevalence when the results suggested obvious publication bias. The prevalence of HCV Ab for each province was also calculated and depicted on the map, using the GIS software.

To assess differences in the accumulation of evidence for HCV Ab prevalence in PWID in Iran, cumulative meta-analyses were conducted. The cumulative meta-analysis provides cumulative pooled estimates and 95% CIs. As studies are successively added, the overall prevalence and 95% CIs are recalculated providing evidence of the evolution of HCV Ab prevalence over time. To assess the sequential contributions of studies and evaluate changes in HCV Ab prevalence over time, studies were added alphabetically by years of implementation to a random-effects model using the “metacum” user-written command in Stata version 16.0. Besides, the sequential contributions of studies were evaluated before 2005 and during and after 2005 for HCV Ab prevalence over time by cumulative meta-analysis.

For investigating the trend of HCV Ab prevalence over time, the pooled prevalence every 3 years was estimated and presented in a line graph because the number of studies conducted in each year was few. The Cochran–Armitage test for linear trend was used by Winpepi software to test the variation in the prevalence of HCV Ab.

## Results

### Study screening and characteristics

A total of 7550 documents were found through searching databases, and 300 were retrieved through other review articles. After removing the duplicates, the titles and abstracts of 5966 documents were screened, of which 5683 documents were excluded because of not meeting the study criteria. If the inclusion criteria were not clear from the abstract, the full text was assessed. In this stage, 221 documents were excluded for different reasons. Finally, data were extracted and subjected to a meta-analysis from the remaining 62 documents (60 cross-sectional studies and 2 case–control studies) (Fig. 1) [15, 21, 42–101].

The prevalence of HCV Ab was assessed in 27,033 PWID in 62 studies. The smallest and largest sample size was 34 and 3,284 PWID, respectively. Twenty-three studies were conducted in DICs, and 16 were done in hospitals and healthcare centers. Table 1 presents the characteristics of the studies. There is a differential distribution of the studies across time by the study recruitment setting (Appendix 3).

**Table 1:** Characteristics of the included studies and the main results

First author and reference	Study date	Study location (city)	Total sample size	Study design	Study sampling procedure	Recruitment setting	HCV Ab prevalence
Alam-Mehrjerdi et al. [100]	2012	Tehran	53	CS	Conv	Drop-in-center and rehab center	43% (30–56%)
Alavi et al. [98]	2002	Ahwaz	104	CS	Conv	Clinical: hospital and healthcare center	74% (65–82%)
Alavi et al. [99]	2003	Ahwaz	142	CS	Conv	Clinical: hospital and healthcare center	52% (43–60%)
Alipour et al. [97]	2013	Mixed (Shiraz, Tehran, and Mashhad)	226	CS	Conv	Drop-in-center and rehab center	38% (32–44%)
Alizadeh et al. [96]	2002	Hamedan	149	CS	SRS	Prison	31% (24–39%)
Amin-Esmaeili et al. [15]	2005	Tehran	895	CS	Conv	Drop-in-centers and rehab center	34% (31–37%)
Aminzadeh et al. [94]	2007	Tehran	70	CS	Conv	Clinical: hospital & healthcare center	36% (24–47%)
Amini et al. [95]	2000	Tehran	34	CS	Conv	Drop-in-center and rehab center	65% (49–81%)
Ahmadzad-Asl et al. [93]	2004	Karaj	150	CS	Conv	Prison	75% (69–82%)
Ataei et al. [92]	2009	Isfahan	3284	CS	Conv	Drop-in-center and rehab center	38% (36–39%)
Ataei et al. [91]	2011	Isfahan	1485	CS	Conv	Prison	43% (40–45%)
Ataei et al. [90]	2011	Isfahan	136	CS	Conv	Drop-in-center and rehab center	19% (13–26%)
Azizi et al. [89]	2008	Kermanshah	263	CS	Conv	Drop-in-center and rehab center	33% (27–38%)
Davoodian et al. [88]	2002	Bandar abbas	249	CS	SRS	Prison	64% (58–70%)
Eskandarieh et al. [87]	2013	Tehran	258	CS	Conv	Drop-in-center and rehab center	65% (60–71%)
Fadaei Nobari et al. [63]	2008	Isfahan	1747	CS	Conv	Community-based	34% (31–36%)
Ghasemian et al. [86]	2009	Sari and Ghaemshahr	88	CS	Conv	Clinical: hospital and healthcare center	38% (27–48%)
Hajinasrollah et al. [85]	2005	Tehran	65	CS	Conv	Clinical: hospital and healthcare center	17% (7–26%)
Honarvar et al. [84]	2012	Shiraz	233	CS	Conv	Drop-in-center and rehab center	40% (34–46%)
Hosseini et al. [83]	2006	Tehran	417	CS	Conv	Prison	80% (76–83%)
Imani et al. [82]	2004	Shahr-e-Kord	133	CS	Conv	Drop-in-center and rehab center	11% (5–16%)
Ismail et al. [81]	2005	Tehran	65	CS	Conv	Clinical: hospital and healthcare center	17% (7–26%)
Kaffashian et al. [80]	2011	Isfahan	951	CS	Conv	Prison	42% (38–45%)
Kassaian et al. [79]	2009	Isfahan	943	CS	Conv	Prison	41% (38–44%)
Keramat et al. [78]	2006	Hamedan	199	CS	Conv	Behavioral consulting center	63% (56–70%)
Khani et al. [77]	2001	Zanjan	346	CS	Conv	Prison	50% (45–56%)
Kheirandish et al. [76]	2006	Tehran	454	CS	Conv	Prison	80% (76–83%)
Khodadadi-zadeh et al. [75]	2003	Rafsanjan	180	CS	Conv	Drop-in-center and rehab center	26% (19–32%)
Khorvash et al. [74]	2005	Isfahan	92	CS	Conv	Clinical: hospital and healthcare center	74% (65–83%)
Majidi et al. [127]	2010	Tehran	104	CS	Conv	Clinical: hospital and healthcare center	7% (2–11%)
Malekinejad et al. [73]	2007	Tehran	564	CS	Respondent-driven sampling	Clinical: hospital and healthcare center	84% (81–87%)
Mehrjerdi et al. [101]	2011	Tehran	209	CS	Conv	Drop-in-center and rehab center	26% (20–32%)
Meidani et al. [72]	2007	Isfahan	150	CS	Conv	Clinical: hospital and healthcare center	26% (19–33%)
Meshkati et al. [71]	2007	Isfahan	98	CS	Conv	Behavioural consulting center	52% (42–61%)
Mir-Nasseri et al. [68]	2002	Tehran	467	CS	NS	Drop-in-center and rehab center	66% (61–70%)
Mir-Nasseri et al. [69]	2002	Tehran	518	CS	Conv	Prison	69% (65–73%)
Mirahmadizadeh et al. [43]	2004	Shiraz	186	CS	Conv	NR	80% (74–85%)
Mirahmadizadeh et al. [70]	2009	Shiraz	1531	CS	SRS	Drop-in-center and rehab center	43% (40–45%)
Mobasheri Zadeh et al. [47]	2011	Isfahan	1055	CS	Conv	Drop-in-center and rehab center	7% (5–8%)
Mohtasham Amiri et al. [67]	2003	Rasht	81	CS	Conv	Prison	88% (82–95%)
Momen-Heravi et al. [66]	2012	Kashan	300	CS	SRS	Drop-in-center and rehab center	47% (41–52%)
Moradi et al. [65]	2015	Iran	678	CS	SRS	Prison	42% (38%–46%)
Naderi et al.[64]	2004	Tehran	144	CS	Conv	Clinical: hospital and healthcare center	22% (15–28%)
Nokhodian et al. [62]	2008	Isfahan	539	CS	Conv	Drop-in-center and rehab center	42% (37–46%)
Noroozi A et al. [42]	2011	Karaj, Isfahan, Gorgan	192	CS	Conv	Drop-in-center and rehab center	28% (21–34%)
Rahbar et al. [61]	2001	Mashhad	101	CC	Conv	Prison	59% (49–69%)
Rahimi-Movaghar et al. [60]	2005	Tehran	899	CS	Snowball sampling	Drop-in-center and rehab center	34% (31–37%)
Ramezani et al. [59]	2012	Arak	100	CS	Conv	Drop-in-center and rehab center	56% (46–65%)
Rezaie et al. [58]	2014	Kermanshah	410	CS	Conv	Drop-in-center and rehab center	42% (37–46%)
Rostami-Jalilian et al. [57]	2003	Isfahan	148	CS	Conv	Clinical: hospital and healthcare center	40% (32–48%)
Saleh et al. [56]	2008	Hamedan	94	CC	Conv	Clinical: hospital and healthcare center	60% (50–70%)
Salehi et al. [55]	2009	Shiraz	1327	CS	Conv	Drop-in-center and rehab center	13% (11–15%)
Sani et al. [49]	2008	Mashhad	62	CS	Conv	Clinical: hospital and healthcare center	71% (59–82%)
Sarkari et al. [54]	2010	Yasuj	158	CS	Conv	NR	42% (34–49%)
Sayad et al. [53]	2006	Kermanshah	1721	CS	SRS	Community-based	50% (47–52%)
Sharhani et al. [52]	2017	Kermanshah	606	CS	Conv	Community-based	55% (51–59%)
Sharif et al. [51]	2004	Kashan	200	CS	Conv	Clinical: hospital and healthcare center	12% (7–16%)
Sofian et al. [50]	2009	Arak	153	CS	Conv	Prison	59% (51–67%)
Tayeri et al. [48]	2004	Isfahan	106	CS	Conv	Clinical: hospital and healthcare center	75% (67–83%)
Zali et al. [46]	1995	Tehran	402	CS	SRS	Prison	45% (40–49%)
Zamani et al. [45]	2004	Tehran	202	CS	Conv	Community-based	52% (45–58%)
Zamani et al. [21]	2008	Isfahan	118	CS	Snowball sampling	Drop-in-center and rehab center	59% (47–69%)

*CS* cross-sectional study, *CC* case–control study, *NR* not reported, *Conv* convenience sampling, *SRS* systematic random sampling

### Risk of bias in studies

The risk of bias and the quality of the study were assessed for the 62 included studies. Most of the studies did not have optimal condition. The highest risk of bias was related to the recruitment setting and sampling method that was seen in most of the studies (Appendix 4).

### Pooled HCV Ab prevalence among PWID and subgroups

**Fig. 2:**
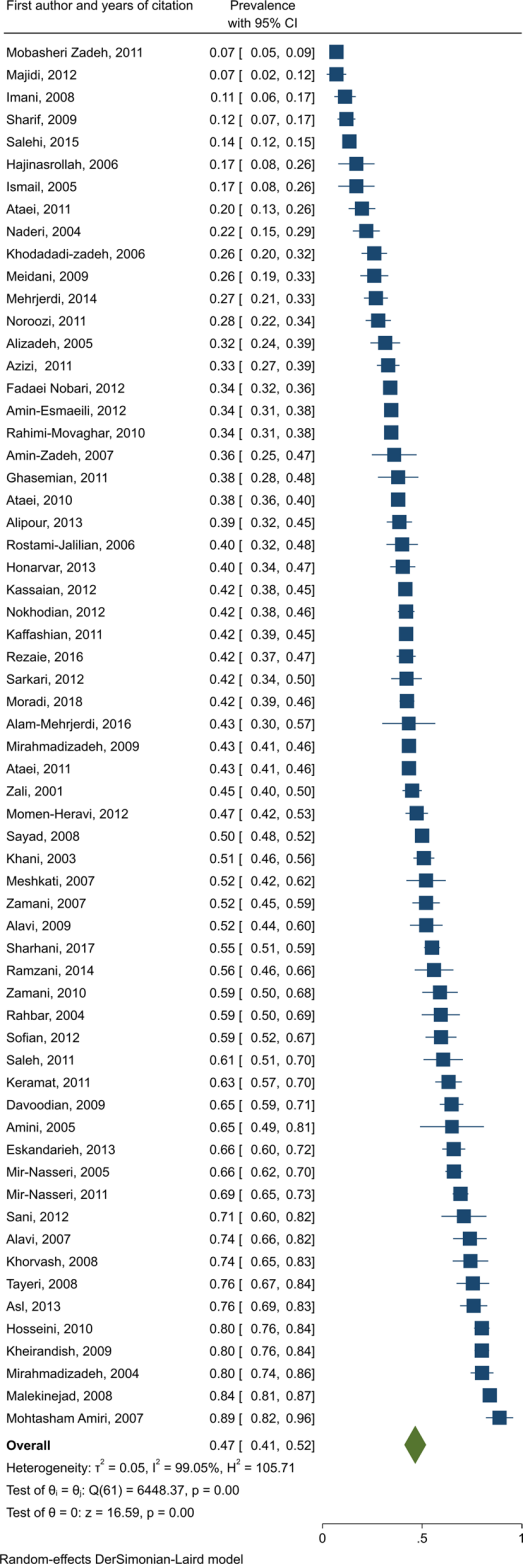
Pooled HCV Ab prevalence in PWID in Iran

**Fig. 3:**
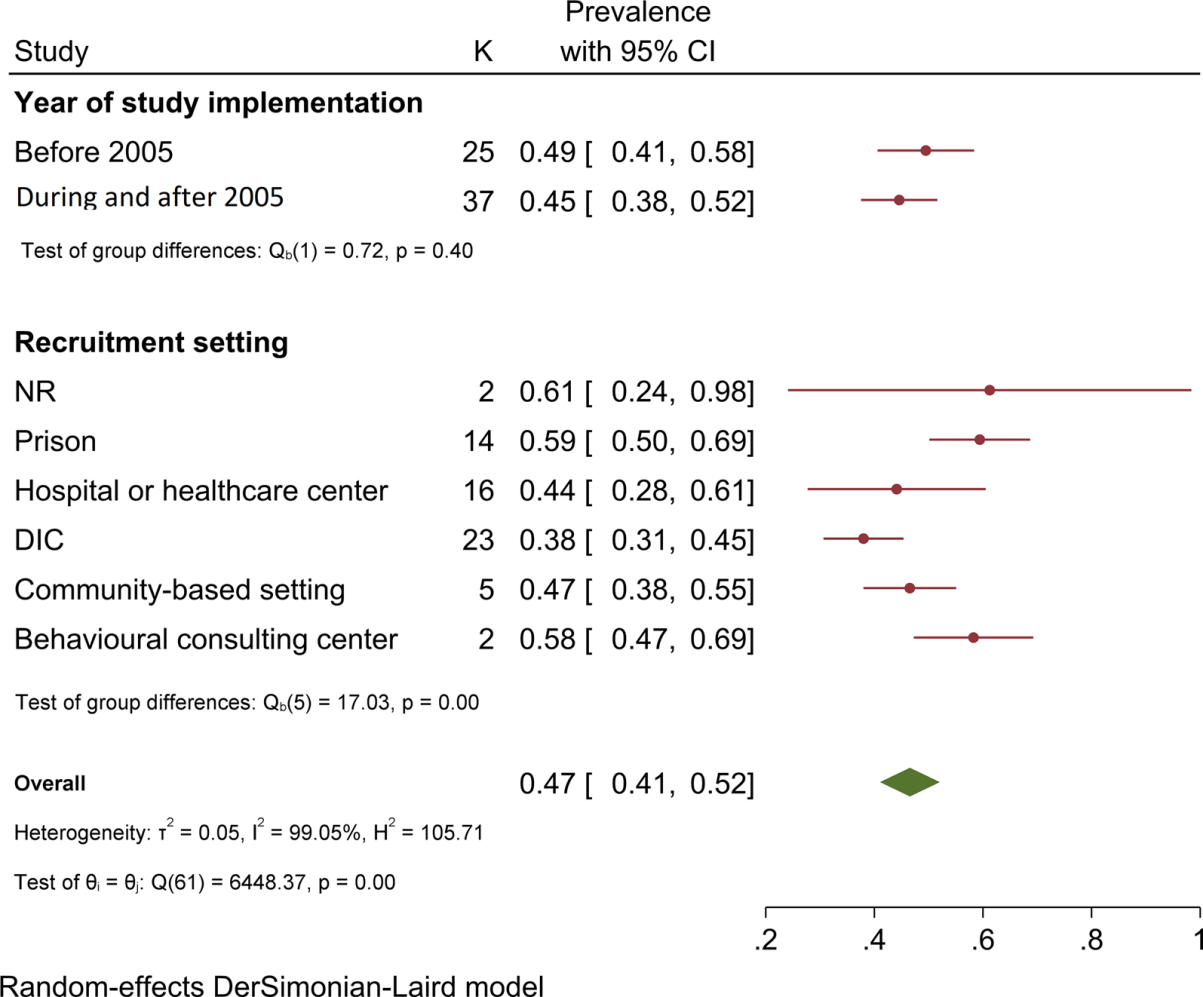
Subgroup analysis of HCV Ab prevalence among PWID in Iran

The pooled HCV Ab prevalence was 46.5% (95% confidence interval [95% CI] 41.1–52.0%), among PWID in Iran (Fig. 2). The pooled prevalence of HCV Ab according to the recruitment setting in a descending order was as follows: prisons [14 studies, N = 6,399, *P* = 59.4%, 95% CI 50.1–68.7%], behavioral disease counseling centers [two studies, N = 297, *P* = 58.3%, 95% CI 47.3–69.3%], community-based settings [five studies, N = 4,954, *P* = 46.5%, 95% CI 38.0–55.1%], hospitals and healthcare centers [16 studies, N = 2,198, *P* = 44.1%, 95% CI 27.7–60.5%], and DICs [23 studies, N = 12,841, *P* = 38.0%, 95% CI 30.6–45.4%] (Fig. 3).

**Fig. 4:**
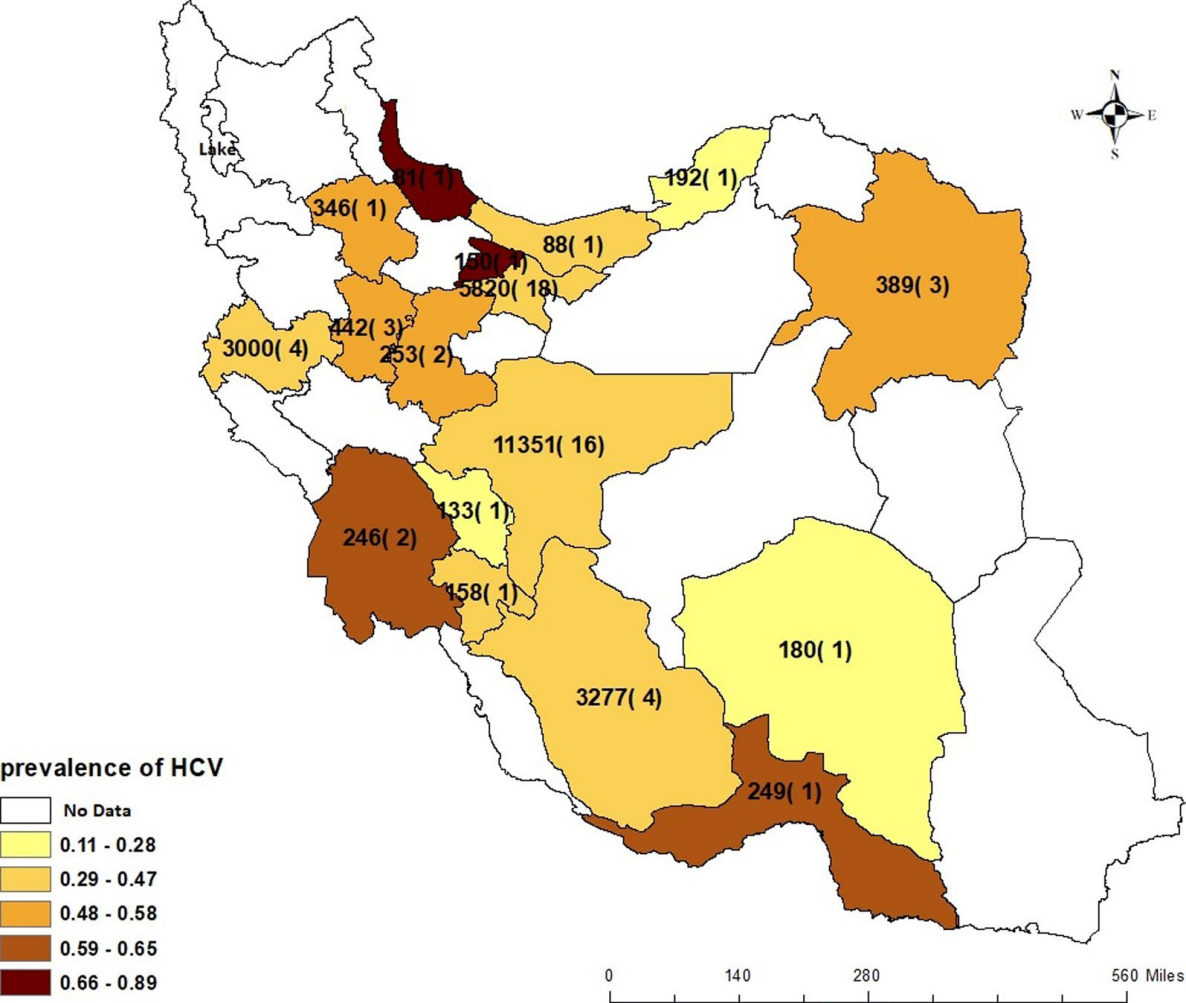
Estimated HCV Ab prevalence in PWID by province (1995–2018). Numbers on each province show the sample size of the studies, and the numbers in the parentheses show the number of studies conducted in that province.

The prevalence of HCV Ab according to the provinces is presented in Fig. 4. The provinces with the highest and lowest HCV Ab prevalence were as follows: Gilan Province (N = 81, *P* = 88.9%, 95% CI 82.1–95.7%) and Chaharmahal Province (N = 133, *P* = 11.3%, 95% CI 5.9–16.7%), respectively. There were no data for 14 provinces (East Azerbaijan, West Azerbaijan, Ardabil, Kurdistan, Qazvin, Lorestan, Bushehr, Ilam, Qom, Semnan, North Khorasan, Yazd, Semnan, South Khorasan, and Sistan and Baluchestan) of Iran. In Tehran, the capital of Iran, and in Isfahan with the highest sample size, the pooled HCV Ab prevalence was 47.0% (95% CI 35.2–58.9%) and 40.6% (95% CI 31.9–49.3%), respectively.

The pooled prevalence of HCV Ab before 2005 and during and after 2005 was 49.5% (95% CI 40.6–58.4%) and 44.6% (95% CI 37.6–51.6%), respectively (test of group differences: Q = 0.7, *P* value = 0.39) (Appendix 5). Pooling the data before harm reduction program (before 2005), the HCV Ab prevalence in prison, hospitals and healthcare centers, and DICs was 61% (95% CI 49–72%), 43% (95% CI 25–61%) and 39% (95% CI 25–53%), respectively. Pooling the data after harm reduction program (during and after 2005), the HCV Ab prevalence in prison, hospitals and healthcare centers, and DICs was 58% (95% CI 43–73%), 46% (95% CI 17–75%) and 38% (95% CI 29–46%), respectively (Appendix 6).

### Cumulative meta-analyses

**Fig. 5:**
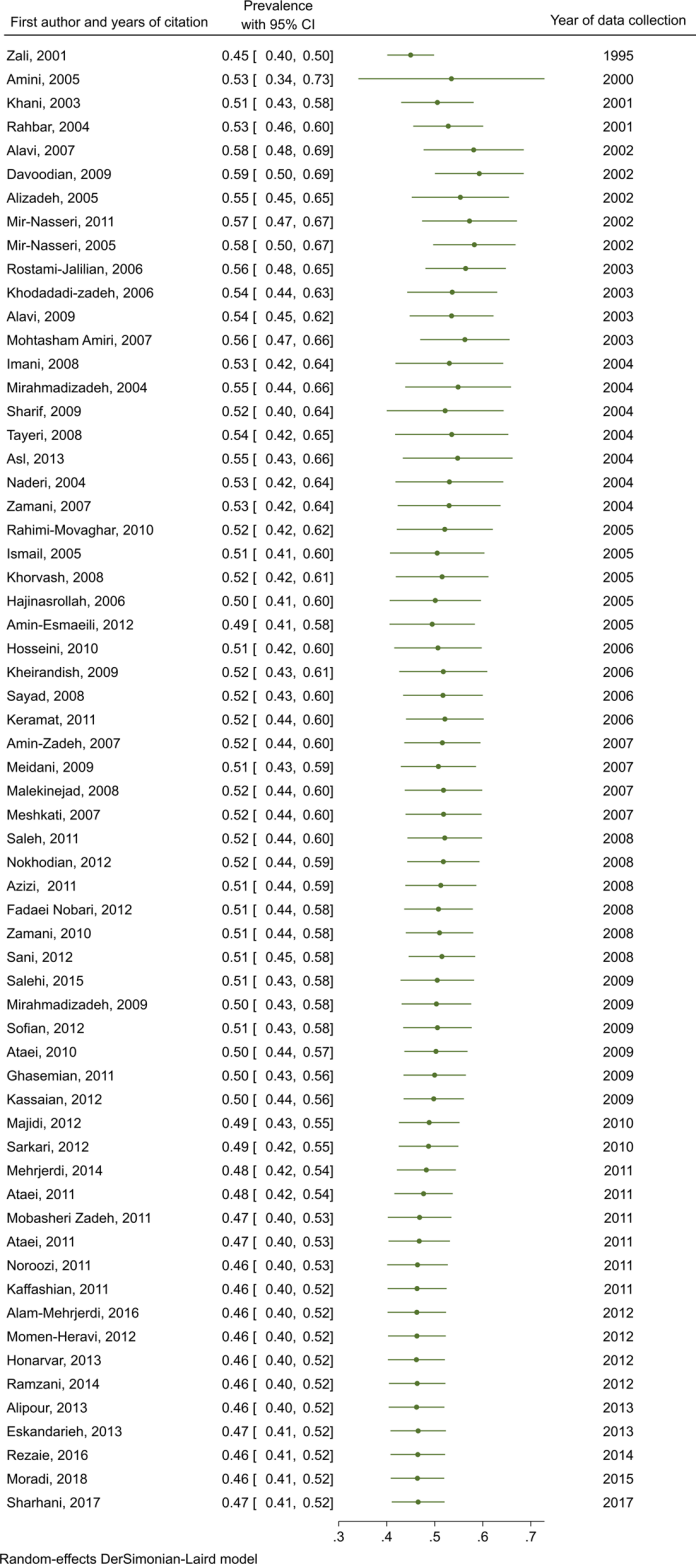
Cumulative meta-analysis of HCV Ab prevalence among PWID in Iran

A cumulative meta-analysis was conducted to reflect the dynamic trend of results and evaluate the influence of individual study on the overall results. Figure 5 shows a forest plot for the cumulative meta-analysis for the trend of HCV Ab prevalence in PWID. A high prevalence (53%) of HCV Ab was first observed in 2000 and remained unchanged or had paradoxical changes after 24 more studies published between 2000 to 2005, and thereafter, the prevalence of HCV Ab between 2005 to 2018 slowly declined (Fig. 5). The cumulative meta-analysis was also presented by the study setting in Appendix 7. The results showed that with an increasing number of studies in later years the prevalence of HCV Ab is somewhat reduced or unchanged in all settings. In the prison setting, the trend of HCV Ab prevalence was increasing by 2002, and then it was slightly declining. This declining trend was also observed for DIC and hospital and healthcare center settings. Besides, considering the potential sampling bias in the hospital setting, we performed the analysis for DIC and prison by the year of study implementation (Appendix 8). The results of this analysis also showed that before 2005, there was no clear trend for the prevalence of HCV Ab. But after 2005, there was a slight decrease until 2011; after that, the trend remained almost stable.

### Trend analysis

**Fig. 6:**
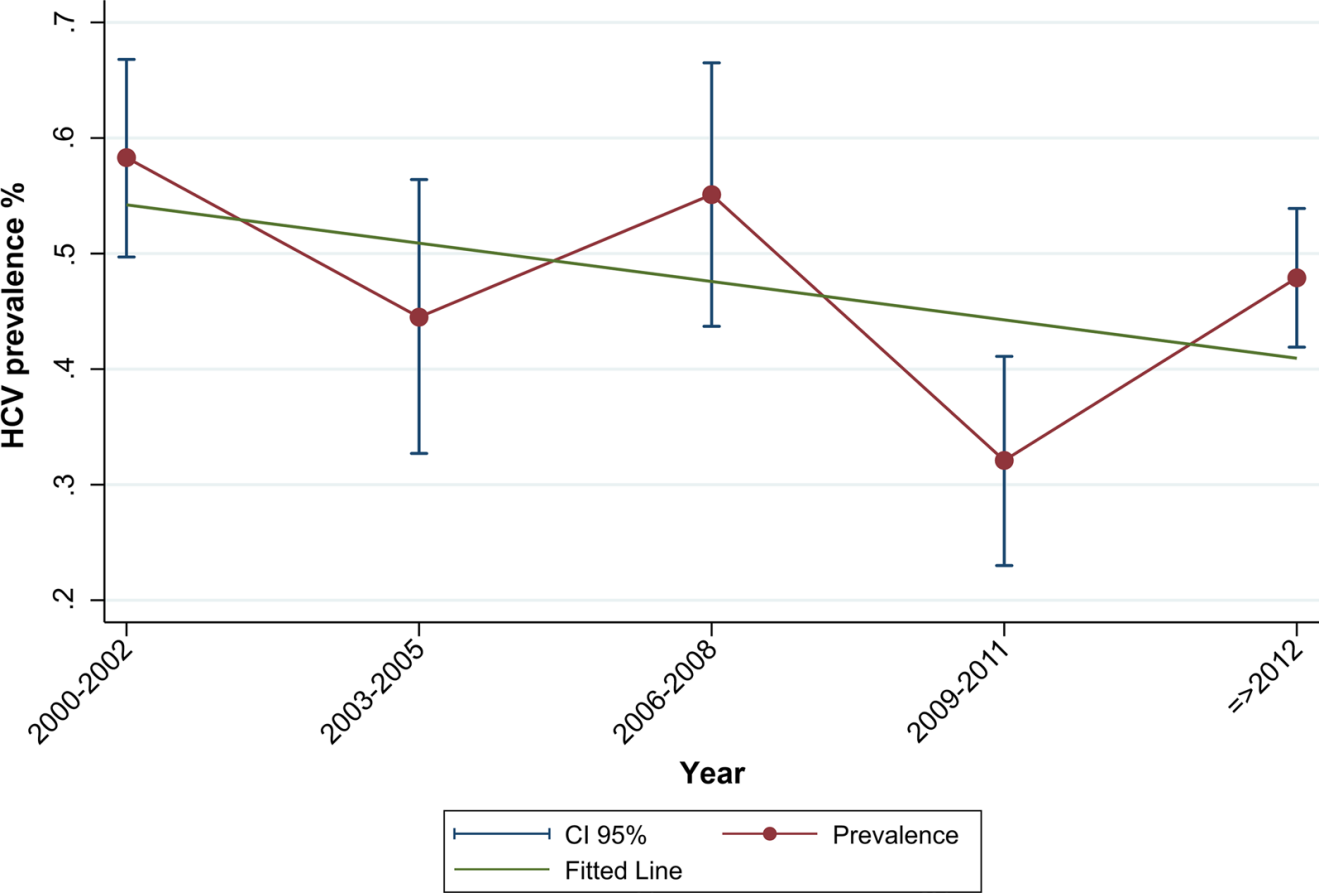
Trend of HCV Ab prevalence among PWID in Iran. The Cochran–Armitage test, *P* value = 0.03

Figure 6 presents the trend of HCV Ab prevalence in PWID in periods from 1995 to 2018. HCV Ab prevalence decreased from 58.3% (95% CI 49.7–66.8%) before 2003 to 44.5% (95% CI 32.7–56.4%) in 2003–2005 and then increased to the level of 55.1% (95% CI 43.7–66.5%) in 2006–2008 and then showed a decline to 32.1% (95% CI 23.0–41.1%) in studies between 2009 and 2011 and then showed a slight increase to the level of 47.9% (95% CI 41.9–53.9%) in studies during and after 2012. In 2009–2011, the decreasing trend of HCV Ab prevalence is statistically significant (b = − 0.23, 95% CI − 0.38, − 0.07, *P* value = 0.004). The Cochran–Armitage test for trend indicated a significant decreasing trend of HCV Ab in PWID in total (*P* value = 0.03).

The trend of HCV Ab prevalence among PWID was presented by the recruitment setting in Appendix 9. The trend of HCV Ab prevalence among PWID in the prison setting did not decrease significantly (Cochran–Armitage test for linear trend = 1.85, *P* value = 0.17). However, trend analysis results indicated a statistically significant decrease in HCV Ab prevalence among PWID in the hospitals and healthcare centers (Cochran–Armitage test for linear trend = 38.72, *P* value < 0.001) and DIC settings (Cochran–Armitage test for linear trend = 5.93, *P* value = 0.01) (Appendix 9).

### Publication bias

**Fig. 7:**
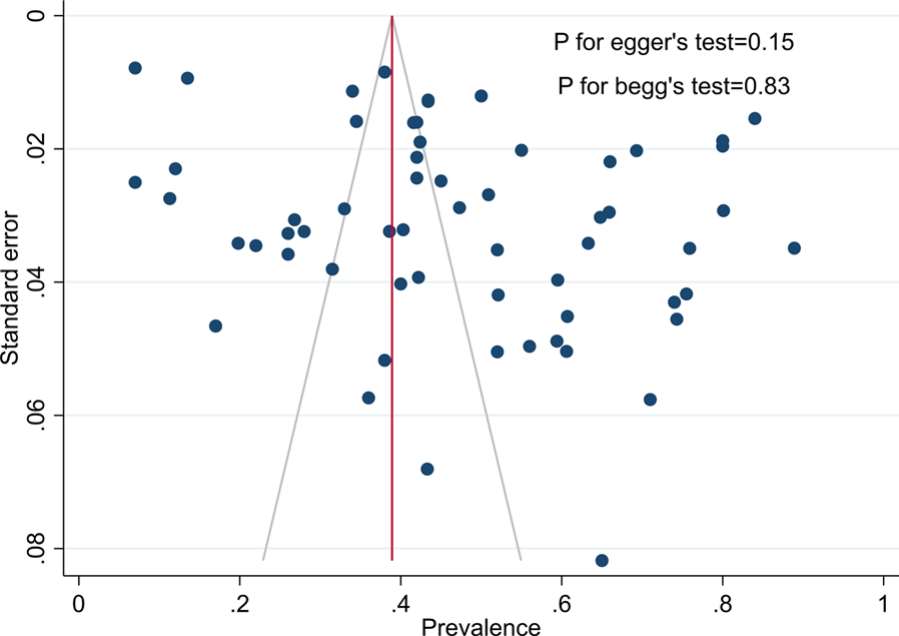
The funnel’s plot for HCV Ab prevalence among PWID in Iran

When we plotted the prevalence estimates against their standard errors, there was no publication bias (Fig. 7). Furthermore, the results were confirmed with Begg and Mazumdar’s tau 0.22 (*P* value = 0.83) and Egger’s regression intercept 1.43 (*P* value = 0.15). Trim-and-fill method for calibration of publication bias was performed. However, the missing study was not identified by the trim-and-fill method.

### Meta-regression analyses

The results of the univariate meta-regression analyses for investigation into the sources of heterogeneity showed that only the recruitment setting had a significant effect on HCV Ab prevalence: date of study (b = − 0.01, 95% CI − 0.02, 0.001, *P* value = 0.08), study type as cross-sectional versus case–control study (b = − 0.13, 95% CI − 0.45, 0.17, *P* value = 0.38), sampling method as random sampling versus convenience sampling (b = 0.001, 95% CI − 0.17, 0.17, *P* value = 0.98), recruitment setting as hospitals and healthcare centers versus prison (b = − 0.15, 95% CI − 0.29, − 0.01, *P* value = 0.03), DIC versus prison (b = − 0.21, 95% CI − 0.34, − 0.08, *P* value = 0.001), and community-based setting versus prison (b = − 0.12, 95% CI − 0.32, 0.06, *P* value = 0.20). Univariate meta-regression showed that the 8.81% of between-study variance is explained by date of study, 0.22% by study design, 0.0% by sampling method, and 21.88% by recruitment setting.

## Discussion

PWID is the main population affected by HCV infection worldwide [102]. The present systematic review and meta-analysis was conducted on 62 observational studies, involving 27,033 PWID. This study examined the prevalence of HCV Ab before and after implementation of the harm reduction program in PWID in Iran. To our knowledge, this is the first systematic review and meta-analysis to examine the prevalence of HCV Ab among PWID before and after implementation of the harm reduction program. In the period since the last major systematic reviews [22, 38], there has been an increase in the amount of evidence documenting drug injection and the prevalence of HCV Ab in PWID in Iran. This updated estimation showed that approximately one in two PWID in Iran are tested positive for HCV Ab, with substantial regional- and provincial-level variations in the prevalence of this blood-borne infection. The results showed a high prevalence of HCV Ab among PWID in Iran, which is consistent with a worldwide estimate in this population [103] and other systematic reviews [22, 38], and still lower than the prevalence reported in certain other countries (e.g., Bulgaria, Estonia, and South Africa) [104, 105] but higher than in studies conducted in Kuwait (12.3%) [106], Kingdom of Saudi Arabia (42.7%) [107], and Brazil (35.6%) [108]. These differences in the prevalence can be attributed to differences in the health systems, screening methods, and the type of high-risk behaviors of individuals [109, 110]. In particular, in the developing countries, the harm reduction programs such as syringe distribution are not fully implemented [111]. Moreover, in the countries with high coverage of HCV care, cases are diagnosed and treated earlier and the transmission of HCV is prevented in the community, which can also be the reason for the inconsistent findings among different studies [112, 113].

The pooled prevalence of HCV Ab was 49% before 2005 and 45% during and after 2005. Evaluation of the trend showed a slightly decreasing trend in the prevalence of HCV Ab in PWID over time. Besides, with the evaluation of the cumulative meta-analysis of HCV Ab prevalence, it seems there was a slightly declined or stable prevalence of HCV Ab among PWID in Iran. The study of Nematollahi et al. [22] showed a decreasing trend in the prevalence of hepatitis C among high-risk groups (β = − 0.021) in 2000–2015. These studies confirmed the findings of the current systematic review, which showed a slight decline in the prevalence of HCV Ab among PWID since 2005.

Globally, studies have shown that implementing NSP and maintenance treatment with methadone reduces high-risk behaviors [30–32]. According to the actions associated with harm reduction in Iran after 2005, this study indicated that the prevalence of HCV Ab among PWID in Iran did not increase. Although the difference in the prevalence before 2005 and after 2005 was not significant, there seemed to be lower prevalence estimates in later years. This study examined the prevalence of HCV Ab which is defined as the number of affected persons with HCV Ab in the population at a specific time [114]. Moreover, when there is a gradual decreasing or stable prevalence of HCV Ab, it is partly attributable to a decrease in the incidence of HCV infection. Thus, the stable or slightly declining prevalence of HCV Ab after 2005 which indicates that new cases of the disease are less reported after 2005 can be partly caused by implementation of the harm reduction program. However, it should be noted that the decrease or stability in the prevalence of HCV Ab may also be influenced by other factors such as the high mortality of PWID with HCV infection, exclusion from being PWID by stopping drug injection and study design differences. Furthermore, another issue that may justify the stable or decreasing prevalence of HCV Ab is that over the past two decades in Iran, drug use patterns changed from traditional drug use, mainly heroin and opium to the recreational use of amphetamine-type stimulants, especially crystal methamphetamine [115, 116]. Several Iranian studies showed that the prevalence of blood-borne infections (including HCV) among people with methamphetamine use is less than those with traditional drug use [39, 117–119]. This is because drug injection or syringe sharing is more in traditional drug users than methamphetamine users [17, 119]. More, the prevalence of HCV Ab is different by the recruitment setting and there is a differential distribution of studies across time by the recruitment setting (e.g., more studies in DICs in recent years) causing a declining trend in the pooled results for HCV Ab prevalence. Therefore, it is needed to be confirmed by a cohort study thoroughly the impact of the harm reduction program on the incidence of HCV infection among PWID. To the best of our knowledge, no study in Iran measured the incidence of HCV infection among PWID. Van Den Berg et al. [34] confirmed in the Amsterdam Addiction Cohort study that the incidence of HCV infection among PWID who received both OST and high coverage NSP was approximately one-third lower than that of those who received either OST or NSP alone. In addition, Sharifi et al. [120] indicated that HIV infection incidence among PWID in 2014 was 5.39 (95% CI 4.71, 6.16) per 1,000 person-years (PY), significantly lower than 17.07 in 2009 (95% CI 15.34, 19.34). Moreover, HIV infection incidence decreased among inmates from 1.34 (95% CI 1.08, 1.67) in 2009 to 0.49 (95% CI 0.39, 0.61) per 1,000 PY in 2013. This study suggested that after an increase in the 2000s, the HIV infection incidence decreased and stabilized among PWID and prisoners in Iran. This could be explained by expanding the preventive interventions, e.g., an increasing number of harm reduction centers for PWID and scaling up free harm reduction services such as increased coverage of the NSP among PWID. The above-mentioned studies suggested that a harm reduction intervention is needed to reach PWID early on to be effective in reducing the risk of HCV transmission. This is especially applicable to Iran if the overall coverage of the NSP and OST programs has reached sufficient among PWID. Other studies have confirmed the effectiveness of the harm reduction program as well [121–124]. In addition, the study by Rahimi et al. [125] reported that the prevalence of HIV was 14.3% before 2007 and 9.7% after 2007. These studies are approving the findings of the current systematic review, which showed sufficient evidence on the effectiveness of harm reduction program in the containment of HCV epidemics among PWID in Iran.

Some limitations of our study were differences in the studies carried out in different provinces and insufficient data from several provinces. The prevalence of HCV Ab in PWID had a wide range in different provinces (11–89%). The regional differences, at least somewhat, might be as a result of variations in time of the study and recruitment settings. It might also be due to differences in socio-demographic characteristics, access conditions for study participants, and high-risk behaviors of PWID in provinces of Iran. In addition, the number of studies conducted in each province is different. In a study [126] conducted in China, the prevalence of HCV Ab was different in various geographical regions. It has been stated that this finding may be due to the differences in the pattern of high-risk behaviors, poverty, and ethnicity in different provinces. These findings indicated that the burden of HCV is still high in some areas, and the scale-up of interventions to prevent and treat HCV among PWID remains a crucial priority to address the HCV epidemics. In the included primary studies, no data were also available about PWID living in the rural areas. Most studies were either conducted in the urban areas or did not report any information about the place of residence (urban versus rural). The reason may be the tendency of PWID to cluster in the cities and urban areas in Iran. Besides, the settings where services for PWID are provided, like DICs, are the main settings for recruitment in the surveys and are mainly located in the cities. However, it causes limitations in the extrapolation of the results to all PWID in the country. Finally, the quality of tests used to determine HCV varied in the included studies and the proportions of the participants receiving the OST and NSP may influence the results of this review; however, we were unable to conduct additional subgroup analysis by the quality of tests and coverage of OST and NSP in this meta-analysis because of the lack of data.

Despite the above-mentioned limitations, the results of this meta-analysis are valuable due to its large sample size, offering evidence to support the hypothesis that after the extensive implementation of harm reduction programs in Iran, the HCV epidemic among PWID has been controlled. We also performed a cumulative meta-analysis by the study date to investigate a trend of the HCV Ab prevalence, and the result indicated that our conclusion was robust when more new studies were added.

## Conclusions

There is a large burden of HCV infection among PWID in Iran. The prevalence of HCV Ab in PWID decreased after 2005, and although not significant, there seemed to be lower prevalence estimates in later years. There are great variations in the prevalence of HCV Ab between different provinces. However, there is no sufficient information available from many provinces. Overall, the results of our study supported the effectiveness of harm reduction program in reducing HCV transmission.

## Figures and Tables

**Fig. 8:**
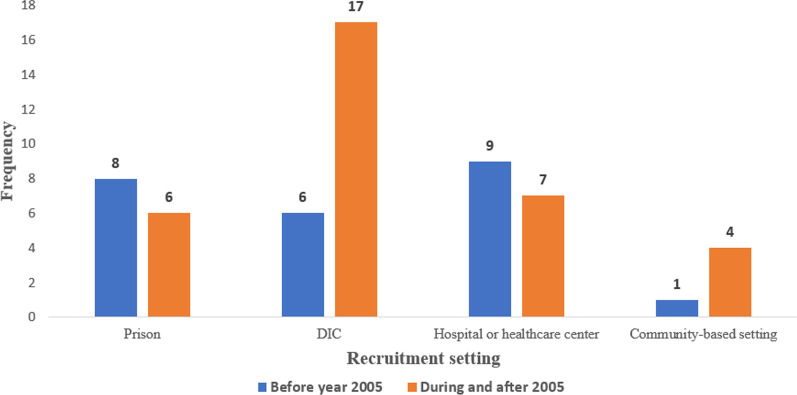
Distribution of studies across time (before 2005 and during and after 2005) by recruitment setting

**Fig. 9:**
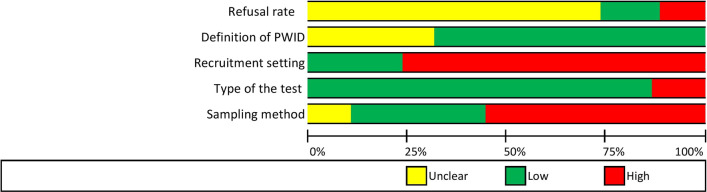
Risk of biases across studies according to the criteria

**Fig. 10:**
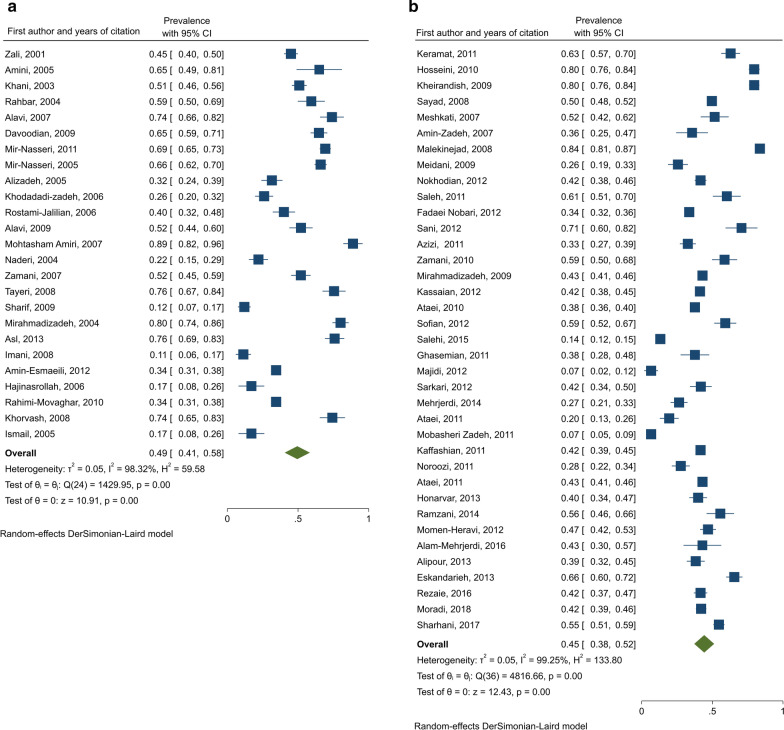
Forest plots for HCV Ab prevalence in PWID by official period of harm reduction. **a** Before 2005; **b** during and after 2005

**Fig. 11:**
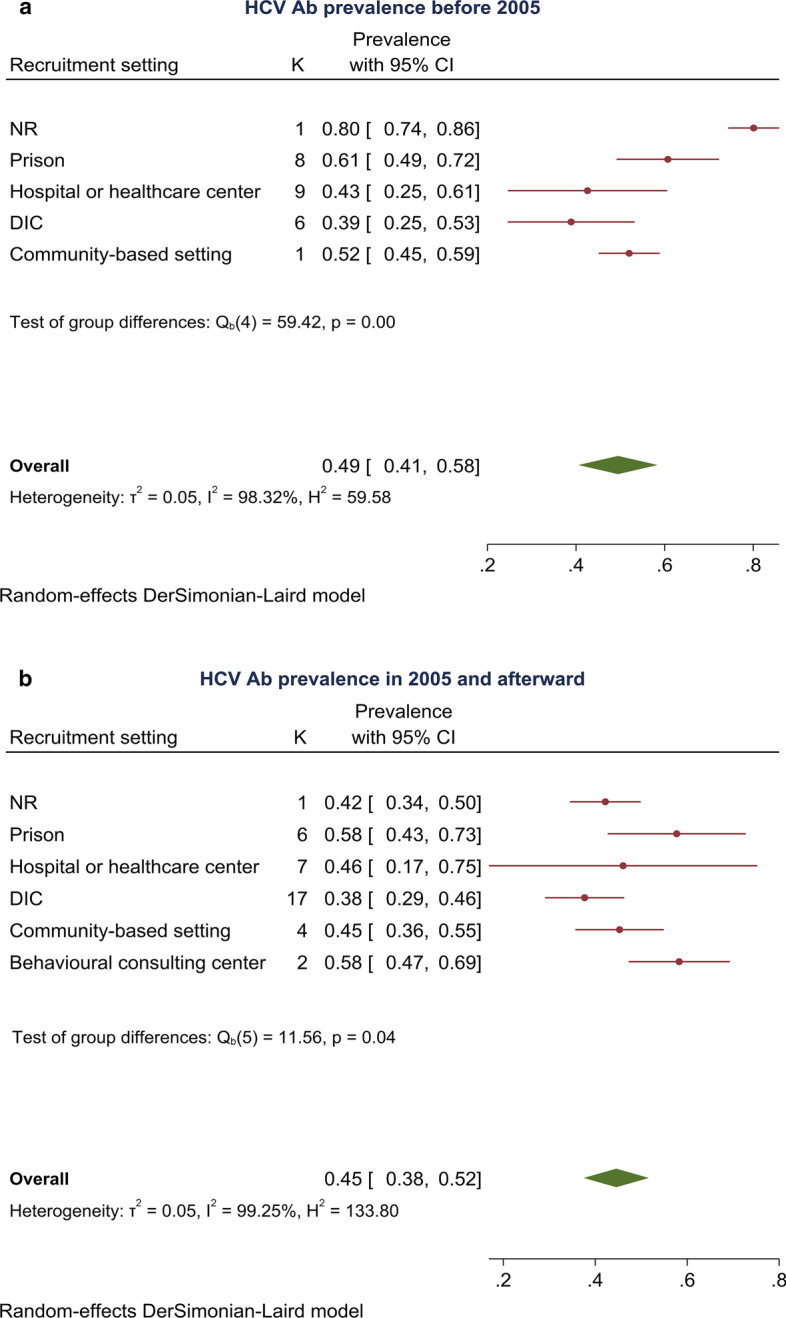
Pooled HCV Ab prevalence among PWID by recruitment setting. **a** Before 2005; **b** during and after 2005

**Fig. 12:**
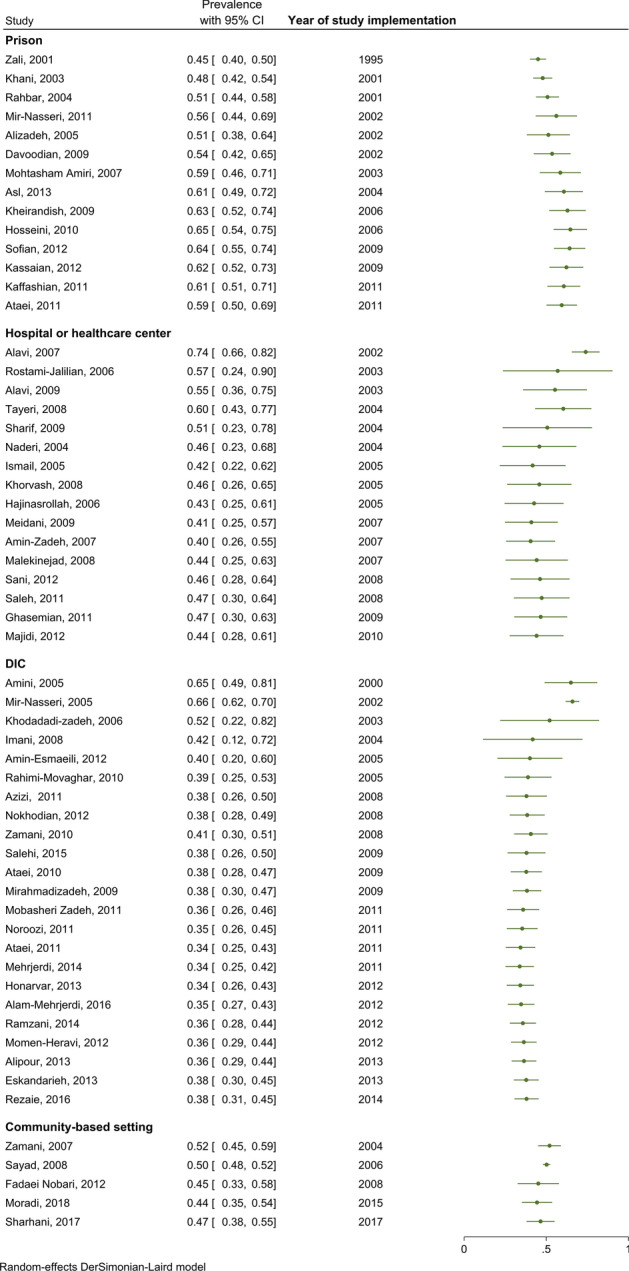
Cumulative meta-analysis of HCV Ab prevalence among PWID in Iran by recruitment setting

**Fig. 13:**
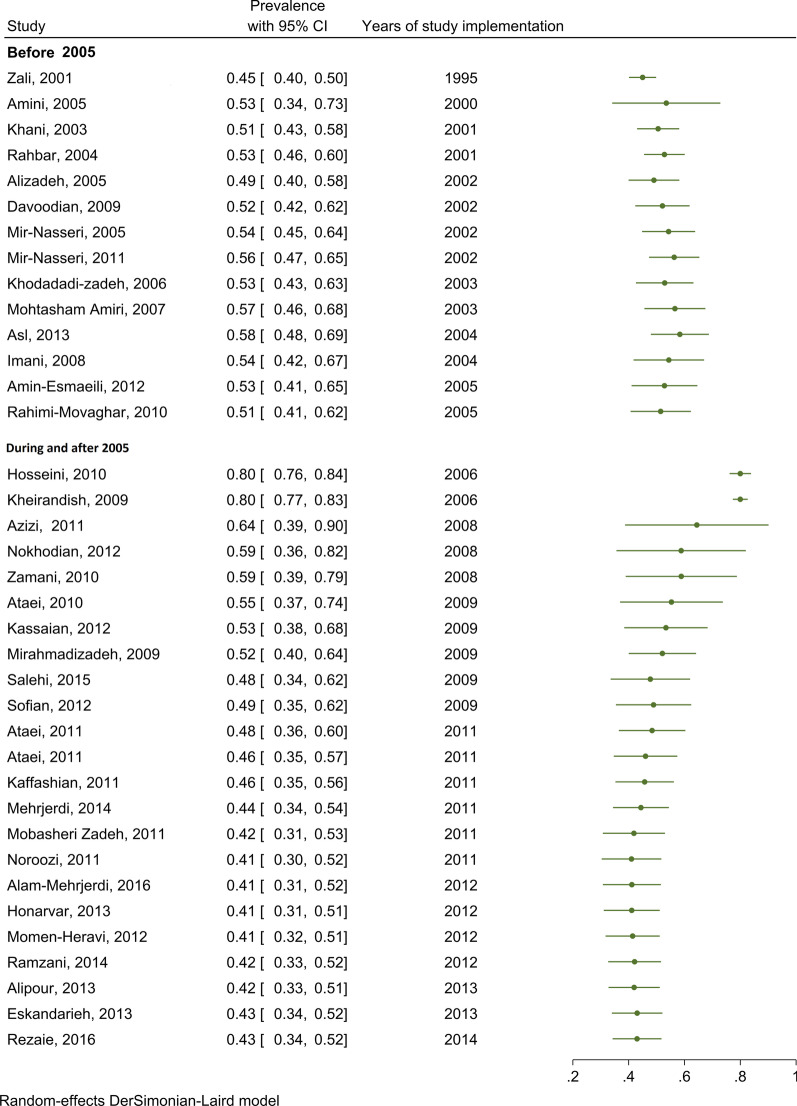
Cumulative meta-analysis of HCV Ab prevalence among PWID in DIC and prison settings in Iran by official period of harm reduction

**Fig. 14:**
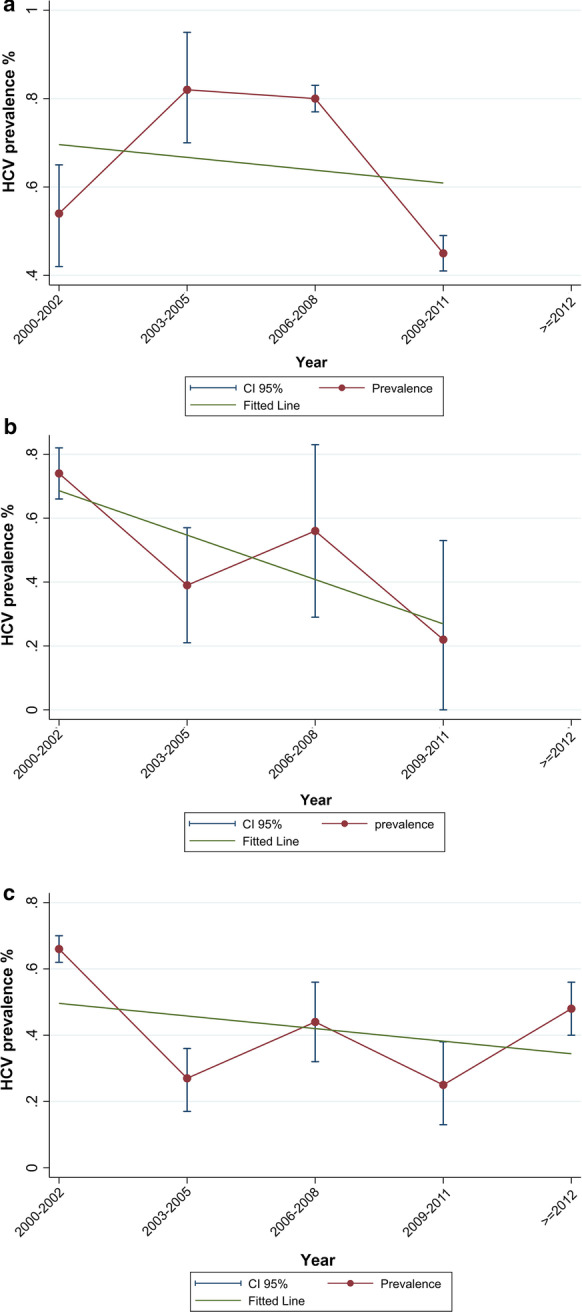
Trend of HCV Ab prevalence among PWID in Iran by recruitment setting. **a** Trend of HCV Ab prevalence among PWID in the prison setting, the Cochran–Armitage test, *P* value = 0.17. **b** Trend of HCV Ab prevalence among PWID in the hospital/healthcare center setting, the Cochran–Armitage test, *P* value < 0.001. **c** Trend of HCV Ab prevalence among PWID in the DIC setting, the Cochran–Armitage test, *P* value = 0.01

## Data Availability

The datasets used and/or analyzed during the current study are available from the corresponding author on reasonable request.

## Appendix 3

See Fig. 8.

## Appendix 4

See Fig. 9.

## Appendix 5

See Fig. 10.

## Appendix 6

See Fig. 11.

## Appendix 7

See Fig. 12.

## Appendix 8

See Fig. 13.

## Appendix 9

See Fig. 14.
